# Antiinfective and wound-healing pleiotropic actions of Ankaferd hemostat

**DOI:** 10.3906/sag-2004-94

**Published:** 2020-08-26

**Authors:** İbrahim Celalettin HAZNEDAROĞLU, Mustafa ÇELEBİER

**Affiliations:** 1 Department of Hematology, Faculty of Medicine, Hacettepe University, Ankara Turkey; 2 Department of Analytical Chemistry, Faculty of Pharmacy, Hacettepe University, Ankara Turkey

**To the Editor,**

We read with great interest the article entitled “An evaluation of the effects of caﬀeic acid phenethyl ester and Ankaferd blood stopper on secondary wound healing of oral mucosal tissue”, published recently in the Journal [1]. The authors indicated that Ankaferd Hemostat (Ankaferd Blood Stopper, ABS) and caffeic acid phenethyl ester had positive eﬀects on the oral wound healing process. Since ABS has already been used in dental surgery for years, it is known as not irritable to the oral mucosa [2]. ABS antibacterial, antiinfective, and cellular features are also established [3]. More importantly, ABS is clinically effective in the prophylaxis and treatment of oral mucositis secondary to chemotherapy and/or radiotherapy in childhood cancers [4] and its safety and efficacy in adult chemotherapy-induced oral mucositis were also reported [5]. From the pharmacological point of view, ABS includes the dried leaf extract (7.0 g/100 mL) of the plant *Glycyrrhiza glabra* [6]. The critical molecular component of *Glycyrrhiza glabra* , glycyrrhizin, has prominent antiinflammatory and antiviral effects [7]. The successful usage of glycyrrhizin in anticancer therapy based on its inhibitory action on the high mobility group box 1 (HMGB1) protein has previously been reported [8]. Moreover, glycyrrhizin is active for the management of SARS-associated virus via inhibiting the replication of the virus [9]. Glycyrrhizin action on SARS virus family could be ascribed to the established pathogenic role of HMGB1 in the SARS disease [10]. Glycyrrhizic acid, present inside *Glycyrrhiza glabra* , also has antiinflammatory effects via inhibiting HMGB1 phosphorylation and secretion [11]. 

 The currently ongoing outbreak of COVID-19, caused by the SARS-CoV-2 coronavirus, enforced worldwide researchers to drug repurposing strategies for identifying new uses of the clinically approved or investigational drugs that are outside the scope of the original medical indication. Some synthetic drugs are already in the progress of repurposing against COVID-19. *Glycyrrhiza glabra* has long been employed against coughs and colds in Chinese Medicine and Wuhan University has proposed a combination of diammonium glycyrrhizinate and vitamin C for COVID-19 therapy [12]. Although herbal medicinal products might be effective against COVID-19, it is hard to suggest them for drug repurposing since most of the plants are not standardized products and not approved by any disease indication. However, ABS is an already approved and standardized plant-based medicinal product for the management of dental, dermal, external and internal bleedings. Its nontoxic and nonirritable features make it possible to be safely applied as topical oral solution [4]. ABS inhibited the growth of nosocomial bacterial pathogens in 72.4% to 100% of the bacteria tested, depending on the type of the isolate [3]. Furthermore, the in vitro yet unpublished results presented that ABS was found to be virucidal on bovine herpes virus type 1 (BHV-1) virus.

Within the light of the recent published paper in Turkish Journal of Medical Sciences [1] regarding the wound healing properties of ABS on oral mucosa and the already published results about the antiinfective effect of ABS, we propose that small-volume ampoule solution of the ABS diluted with water shall be used as a gargling solution for the virus-induced oropharyngeal mucositis, clinically representing itself as ‘sore throat’. The antimicrobial, antiinfective, virucidal, antiseptic and wound healing features of ABS [3] could be beneficial for the clinical management of initial steps of COVID-19 infection for instance at the oropharyngeal level. Repurposing of the topical oral usage of ABS against COVID-19 may be tested in clinical trials in near future for this aim of repositioning. 
